# Cruciate Ligament Reconstruction and Risk of Knee Osteoarthritis: The Association between Cruciate Ligament Injury and Post-Traumatic Osteoarthritis. A Population Based Nationwide Study in Sweden, 1987–2009

**DOI:** 10.1371/journal.pone.0104681

**Published:** 2014-08-22

**Authors:** Richard Nordenvall, Shahram Bahmanyar, Johanna Adami, Ville M. Mattila, Li Felländer-Tsai

**Affiliations:** 1 Division of Orthopedics and Biotechnology, Department of Clinical Science, Intervention and Technology, Karolinska Institutet, Stockholm, Sweden; 2 Department of Orthopedics, Karolinska University Hospital, Stockholm, Sweden; 3 Clinical Epidemiology Unit, Department of Medicine, Karolinska Institutet, Stockholm, Sweden; 4 Center for Pharmacoepidemiology, Department of Medicine, Karolinska Institutet, Stockholm, Sweden; 5 Faculty of Medicine, Golestan University of Medical Sciences, Gorgan, Iran; 6 Department of Orthopedics and Trauma Surgery, University Hospital of Tampere, Tampere, Finland; University of Sheffield, United Kingdom

## Abstract

**Objective:**

To study the association between Cruciate Ligament (CL) injury and development of post-traumatic osteoarthritis in the knee in patients treated operatively with CL reconstruction compared with patients treated non-operatively.

**Design:**

Population based cohort study; level of evidence II-2.

**Setting:**

Sweden, 1987–2009.

**Participants:**

All patients aged between 15–60 years being diagnosed and registered with a CL injury in The National Swedish Patient Register between 1987 and 2009.

**Main Outcome Measures:**

Knee osteoarthritis.

**Results:**

A total of 64,614 patients diagnosed with CL injury during 1987 to 2009 in Sweden were included in the study. Seven percent of the patients were diagnosed with knee OA in specialized healthcare during the follow-up (mean 9 years). Stratified analysis by follow-up showed that while those with shorter follow-up had a non-significant difference in risk (0.99, 95%CI 0.90–1.09 for follow-up less than five years compared with the non-operated cohort), those with longer follow-up had an increased risk of knee OA after CL reconstruction (HR = 1.42, 95%CI 1.27–1.58 for follow-up more than ten years compared with non-operated cohort). The risk to develop OA was not affected by sex.

**Conclusion:**

CL reconstructive surgery does not seem to have a protective effect on long term OA in either men or women.

## Introduction

Musculoskeletal injuries are common worldwide [1]. A predominant location is the knee joint, where the cruciate ligaments play a vital role in both stabilization and kinematics [2].

Injuries to the cruciate ligaments (CL) are common and generally affect the anterior cruciate ligament (ACL) [3]. These injuries occur primarily in activity with knee-pivoting movements such as soccer, basketball and alpine skiing. The mean age at time of diagnosis is 32 years [3]. The incidence of diagnosed CL injury in Sweden is 78 per 100,000 inhabitants and approximately 36% undergo reconstructive surgery [3]. Although men have an increased risk for CL injury in the general population (RR = 1.44) compared with women [3], the risk among women participating in certain sports is between 2–9 times higher than the risk among men participating in the same activities [3]–[9].

The most important complication after ACL injury is knee osteoarthritis (OA). In the general population OA in the knee has a reported prevalence of 5% in patients over 26 years and 12% in patients over 60 years [10]–[12]. Known risk factors for OA are age, obesity and knee trauma [13]. CL injury increases the risk for OA and the prevalence of knee OA after CL injury varies in different studies. Results from a meta-analysis and two systematic reviews show a prevalence of OA after CL injury ranging between 0–48% [14]–[16]. Apart from well-established risk-factors in the CL-sufficient knee the presence of associated injuries such as meniscus and cartilage injuries increase the risk for developing OA after CL-injury. A concomitant meniscal tear occurs in 25–65% of the cases [17].

Optimal treatment of a CL injury is under continuous debate and new inventions range from a multitude of surgical methods, fixation devices and rehabilitation protocols [18], [19]. A number of different techniques and grafts have been suggested [20]–[22]. The purpose of CL reconstruction (CL-R) is to counteract knee instability and to restore kinematics, aiming to facilitate return to a desired activity level (often including pivoting sports) regardless of the risk to develop OA. Although treatment of CL injury varies in different countries reconstructive surgery of the CL is considered as the first line of treatment for specific groups of patients such as elite athletes, while conservative treatment with structured rehabilitation is considered to have a corresponding outcome in the general population [23], [24]. Some studies have reported that CL-R decreases the risk of post traumatic OA. However the results are conflicting [14], [25], [26] and the studies have limitations such as small sample sizes or short follow-up time.

This nationwide cohort study with long follow-up time used data from the National Swedish Patient Register to estimate the risk of OA in the knee after CL injury for patients treated surgically compared to patients treated non-surgically.

## Methods

### Ethics statement

All registry information was anonymized and de-identified by the Swedish National board of Health and Welfare prior to analysis. This study was approved by the regional Ethics Committee at Karolinska Institutet (Dnr: 2010/1713-32).

### Data from the Swedish National Patient Register

The study is a nationwide, open cohort study using data from the Swedish National Patient Register (NPR) [27]. This register was established in 1964 by the Swedish National board of Health and Welfare and has national coverage for all inpatient care since 1987. Information of outpatient care, including information on ambulatory care at hospitals, has been recorded since 2001. Each record in the NPR, corresponding to one hospital-episode, contains the date for hospital admission and discharge, age, sex, hospitals code, clinical ward, surgical procedures and up to eight discharge diagnoses coded according to the International Classification of Disease (ICD-7 until 1968, ICD-8 1969–1986, ICD-9 1987–1996 and ICD-10 thereafter). The national registration number, a unique identifier assigned to all Swedish citizens, allows linkage of data. Visits in primary healthcare, i.e. healthcare by general practitioners, are not included in the Swedish National Patient Register.

### Identifying cruciate ligament reconstruction and knee osteoarthritis

We included all patients diagnosed with CL injury (ICD-9: 8442 – Cruciate ligament in knee, ICD-10: S835 – Distortion engaging the cruciate ligament in the knee, S837 – Injury to multiple structures of knee, M235 – Chronic instability in the knee-joint) for the first time between 1987 and 2009 (n = 84,358). We excluded patients younger than 15 years at the time of diagnosis (n = 3,224) since ACL injury among children with open epiphysis differ regarding treatment and outcome from adults. We also excluded patients over 60 years since CL injuries in older patients are relatively rare and we expect many of these cases to be misclassified (n = 1,470).

NPR was also used to identify those who underwent CL reconstructive surgery (the Swedish version of Classification of Surgical Procedures (NOMESCO): NGE41, NGE42, NGE49, NGE51, NGE52). Our main outcome variable was diagnosis of OA (patients with a diagnosis of knee OA (ICD-10 codes M170-M179 and corresponding ICD-9 codes) and those undergoing operations due to knee OA such as osteotomy, prosthesis etc. (surgical procedures code 8191, 8423–8428, NGB09-NGB99, NGC09-NGC99, NGG09-NGG99, NGK59, NGN49; NGU09, NGH2)).

A registered meniscus injury (ICD-10 codes M232, M233, S832, S837 and corresponding ICD-9 codes) or meniscus surgery (surgery code NGD) was classified as an acute meniscus injury if the patient was 35 years old or younger and was included as a confounding factor. By using this age limitation we aimed to exclude the majority of degenerative meniscal injuries which are not confounders but early signs of osteoarthritis and which commonly are miscoded as acute ruptures instead of degenerative meniscal injuries [28]. A meniscal injury was included as a confounding factor independent of the relationship between the date of meniscal injury/surgery and the date of the CL injury diagnosis.

Dates of emigration and death for the entire study period were obtained using the Swedish Total Population Register.

### Statistical analysis

Cox proportional hazard model was used to estimate hazard ratios (HR) with 95% confidence intervals (CI) as a measurement for the association between CL-R and development of knee OA where CL-R was treated as time-varying covariate. Follow-up started from the date of registered CL injury until date of diagnosis of knee OA or operation due to knee OA, emigration, death or December 31^st^ 2009, whichever came first. We excluded all patients with a follow-up shorter than two years since OA prevention trials need a follow up time of at least two years (n = 15,050). The models were internally stratified for calendar (year of entry into the cohort) category of age (<20, 20–25, 25–30, 30–35, 35–40, 40–45, 45–50 and >50 years) and sex. In Model 2 we also adjusted for meniscal injury (a registered diagnosis of meniscal injury or meniscal surgery). As the time from CL injury to CL-R might be important, stratified analysis were performed for this covariate (categorized to: <3 months, 3 months to one year, >1 year). We estimated the follow-up specific relative risks (follow-up 2–4,9 years, 5–9,9 years, >10 years). As information of outpatient care has been recorded since 2001, we also estimated the relative risks for patients being diagnosed before and after 2001.

To check for interaction effects, we added interaction terms of CL-R and meniscal injury (a registered diagnosis of meniscal injury or meniscal surgery) into the full models.

All statistical analyses were performed using SAS (Statistical Analysis Software, version 9.2, SAS Institute Inc).

## Results

A total of 64,614 patients diagnosed with CL injury during 1987 to 2009 were included in the analysis. Men represented 63% and the mean age at time of diagnosis was 29 years (range 15–60, SD = 10). In total, 48% went through reconstructive surgery, and 41% of the patients had a traumatic meniscus injury (Table 1). In the group treated surgically the mean age at the time of diagnosis was 26 years (range 15–60, SD = 8) compared with 32 years (range 15–60, SD = 11) in the group treated non-operatively. In total 7% (4,314) of the patients were diagnosed with knee OA. Of those 10% (444) underwent osteotomy or either partial or total knee replacement.

**Table 1:** Characteristics of the study population according to type of treatment and follow-up time.

	Number of subjects	Male (%)	Meniscal-injury (%)	Mean age at CL injury (SD)	Mean age at end of follow-up (SD)
**Total**	**64614**	**40398 (63%)**	**26797 (41%)**	**29.15 (10,32)**	**38.15 (11.09)**
No CL-R	33695	21036 (62%)	11027 (33%)	32.03 (11.18)	40.75 (11.80)
CL-R	30919	19362 (63%)	15770 (51%)	26.01 (8.22)	35.31 (9.47)
**Follow up 2–4,9 years**					
No CL-R	10260	6186 (60%)	3371 (33%)	33.04 (11.90)	36.55 (11.93)
CL-R	7644	4516 (59%)	4200 (55%)	26.14 (9.33)	29.63 (9.38)
*- CL-R within 3 months*	2868	1701 (59%)	1342 (47%)	26.15 (9.28)	29.60 (9.32)
*- CL-R between 3 months and 1 year*	3328	1962 (59%)	1929 (58%)	25.92 (9.26)	29.38 (9.32)
*- CL-R after 1 year*	1448	853 (59%)	929 (64%)	26.61 (9.60)	30.26 (9.62)
**Follow up 5–9,9 years**					
No CL-R	13465	8433 (63%)	4554 (34%)	32.82 (11.14)	40.07 (11.19)
CL-R	11335	7068 (62%)	6283 (55%)	26.51 (8.45)	33.96 (8.59)
*- CL-R within 3 months*	4905	3052 (62%)	2296 (47%)	26.50 (8.28)	34.26 (8.44)
*- CL-R between 3 months and 1 year*	3537	2199 (62%)	2065 (58%)	26.35 (8.55)	33.49 (8.66)
*- CL-R after 1 year*	2893	1817 (63%)	1922 (67%)	26.73 (8.59)	34.04 (8.72)
**Follow up >10 years**					
No CL-R	9970	6417 (64%)	3102 (31%)	29.93 (10.15)	45.99 (10.43)
CL-R	11940	7778 (65%)	5287 (44%)	25.44 (7.13)	40.23 (7.70)
*- CL-R within 3 months*	9243	6055 (65%)	3632 (39%)	25.53 (7.10)	40.47 (7.72)
*- CL-R between 3 months and 1 year*	1105	683 (62%)	658 (60%)	25.42 (7.18)	38.51 (7.38)
*- CL-R after 1 year*	1592	1040 (65%)	997 (63%)	24.96 (7.24)	40.01 (7.62)

The mean follow-up time for the entire study population was 9 years (range 2–23, SD = 5) and 8 years (range 2–23, SD = 5) for those with an event of OA. The mean delay between CL diagnosis and CL-R was 266 days (range 0–8,346, SD = 598).

There was no statistically significant difference in the risk of OA among males and females (HR 1.03, 95%CI 0.96–1.09). Having a concomitant meniscal injury increased the risk of OA (HR 2.94, 95%CI 2.72–3.17). There was a statistically significant interaction between CL-R and meniscal injury with respect to OA risk (p = 0.01). Stratified analysis by meniscal injury showed no evident difference in risk for those treated surgically compared with those treated non-operatively (HR 1.22 95%CI 1.12–1.33 HR for those with meniscal injury and 1.21 95%CI 1.10–1.33 for those without meniscal injury) when adjusted for sex, age and year of entry in the cohort (Table 2). There was no significant difference in risk to develop OA in patients treated surgically compared with those treated non-surgically when comparing patients being diagnosed before and after 2001.

**Table 2:** Hazard ratio (HR) and 95% confidence interval (CI) for the association between crucial ligament reconstruction and knee osteoarthritis, according to meniscal injury and follow-up.

	Number of events	Adjusted HR (95% CI)*
**Overall**		
No CL-R	2199	Reference
CL-R	2115	1.26 (1.18–1.34)
- No meniscal injury	780	1.21 (1.10–1.33)
- Meniscal injury	1335	1.22 (1.12–1.33)
- Meniscal injury without surgery	263	1.29 (1,07–1,57)
- Meniscal injury with surgery	1072	1,17 (1,061,29)
**Follow up 2–4,9 years**		
No CL-R	925	Reference
CL-R	630	1.05 (0.95–1.16)
- No meniscal injury	261	1.04 (0.89–1.22)
- Meniscal injury	369	1,01 (0.85–1.18)
- Meniscal injury without surgery	73	0.94 (0.70–1.26)
- Meniscal injury with surgery	296	0.97 (0.83–1.12)
**Follow up 5–9,9 years**		
No CL-R	638	Reference
CL-R	731	1.29 (1.16–1.44)
- No meniscal injury	263	1.12 (0.94–1.33)
- Meniscal injury	468	1.10 (0.94–1.29)
- Meniscal injury without surgery	82	1.55 (1.12–2.15)
- Meniscal injury with surgery	386	1.21 (1.04–1.41)
**Follow up >10 years**		
No CL-R	636	Reference
CL-R	754	1.43 (1.28–1.60)
- No meniscal injury	256	1.42 (1.20–1.70)
- Meniscal injury	498	1.41 (1.22–1.63)
- Meniscal injury without surgery	108	1.63 (1.18–2.23)
- Meniscal injury with surgery	390	1.42 (1.21–1.67)

*The models were internally stratified for sex, age-group and calender.

**Figure 1:**
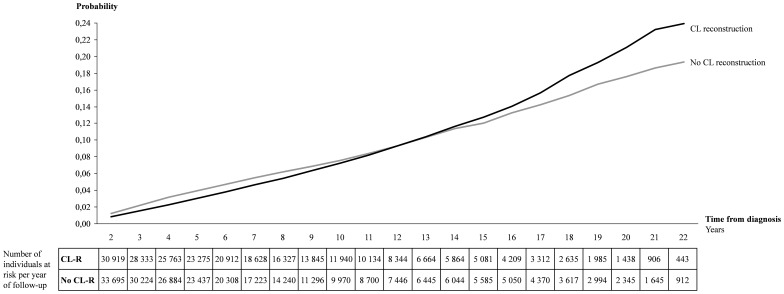
Cumulative incidence for osteoarthritis in the knee among 33,695 patients with CL diagnosis treated non-operatively and 30,919 patients with CL diagnosis treated surgically in Sweden 1987–2009.

During the first years of follow-up, the cumulative incidence of OA was higher among those without CL-R and the trend changed after ten years follow-up (Figure 1). Approximately 24% of those with CL-R were diagnosed with OA at the end of follow-up and the corresponding rate was approximately 19% among those who did not undergo CL-R.

In the overall analysis an increased risk for OA in the knee was observed in patients with CL injury who were treated surgically compared with those treated non-surgically (HR = 1,22, 95%CI 1,14–1,30). Stratified analysis by follow-up showed that while those with shorter follow-up showed no significant difference in risk (HR 0.99, 95%CI 0.90–1.09 for follow-up less than five years) those with longer follow-up had an increased risk of knee OA after CL-R (HR = 1.42, 95%CI 1.27–1.58 for follow-up more than ten years). Stratified analysis by time of operation since diagnosis for each strata of follow-up time did not attenuate the relative risks notably (Table 3).

**Table 3:** Hazard ratio (HR) and 95% confidence interval (CI) for the association between crucial ligament reconstruction and knee osteoarthritis, according to follow-up and time of surgery.

	Number of events	Crude HR (95% CI)	Adjusted HR (95% CI)	
			Model 1*	Model 2**
**Overall**				
No CL-R	2199	Reference	Reference	Reference
CL-R	2115	1.05 (0.99–1.12)	1.26 (1.18–1.34)	1.22 (1.14–1.30)
- CL-R within 3 months	1419	1.00 (0.94–1.07)	1.21 (1.12–1.29)	1.23 (1.15–1.32)
- CL-R between 3 months and 1 year	364	1.17 (1.04–1.31)	1.40 (1.24–1.57)	1.24 (1.11–1.40)
- CL-R after 1 year	332	1.15 (1.03–1.29)	1.36 (1.21–1.53)	1.13 (1.00–1.27)
**Follow up 2–4,9 years**				
No CL-R	925	Reference	Reference	Reference
CL-R	630	0.84 (0.77–0.93)	1.05 (0.95–1.16)	0.99 (0.90–1.09)
- CL-R within 3 months	322	0.68 (0.60–0.77)	0.86 (0.75–0.98)	0.88 (0.77–1.01)
- CL-R between 3 months and 1 year	192	1.00 (0.86–1.16)	1.25 (1.07–1.45)	1.15 (0.99–1.34)
- CL-R after 1 year	116	1.04 (0.89–1,20)	1.20 (1.04–1.39)	1.03 (0.89–1.19)
**Follow up 5–9,9 years**				
No CL-R	638	Reference	Reference	Reference
CL-R	731	1.11 (1.00–1.23)	1.29 (1.16–1.44)	1.26 (1.13–1.40)
- CL-R within 3 months	494	1.07 (0.95–1.19)	1.27 (1.13–1.44)	1.33 (1.18–1.50)
- CL-R between 3 months and 1 year	123	1.23 (1.00–1.50)	1.35 (1.10–1.66)	1.20 (0.98–1.48)
- CL-R after 1 year	114	1.23 (0.98–1.53)	1.33 (1.07–1.66)	1.08 (0.87–1.35)
**Follow up >10 years**				
No CL-R	636	Reference	Reference	Reference
CL-R	754	1.31 (1.18–1.46)	1.43 (1.28–1.60)	1.42 (1.27–1.58)
- CL-R within 3 months	603	1.30 (1.16–1.45)	1.41 (1.26–1.58)	1.44 (1.29–1.61)
- CL-R between 3 months and 1 year	49	1.52 (1.12–2.07)	1.72 (1.26–2.35)	1.40 (1.02–1.91)
- CL-R after 1 year	102	1.32 (0.99–1.76)	1.49 (1.11–2.00)	1.21 (0.90–1.62)

*The models were internally stratified for sex, age-group and calender.

**Additionally adjusted for meniscal injury.

## Discussion

To the best of our knowledge this is the first population based nationwide study describing the association between CL injury and the development of knee OA in a large cohort. We observed that surgically reconstructed ACL injured patients were more frequently diagnosed with knee OA in specialized healthcare during follow-up than non-reconstructed patients. This suggests that decreasing the long-term risk for post traumatic OA after CL injury is not an argument for CL reconstruction. The results also demonstrated that the risk to develop OA did not differ between males and females. Concomitant meniscal injury was associated with increased risk for OA irrespective of timing of surgery.

The strengths of this study include the large nationwide patient database with excellent coverage [29], equal access to health care in Sweden and relatively long follow-up allowing for analysis of the development of OA in both patients treated conservatively and surgically. Moreover, since we had the opportunity to control for major confounding factors included in the registry and this is a cohort with prospectively collected outcome data the possibility of bias is limited. However, possible selection bias cannot be completely ruled out since more extensive knee trauma increases the risk for osteoarthritis and might as well be associated with a higher risk to be selected for surgery. The risk for reverse causality is small since the main symptom for osteoarthritis is pain and according to Swedish protocols pain has never been an indication for CL reconstruction. The result of stratified analysis showed that the risk for osteoarthritis increased with increasing time after surgery which also strengthens this argumentation.

The main limitation of this study is potential misclassification of CL-injury, OA and acute meniscus injury since the registry does not include information about criteria or diagnostic methods. CL injury is diagnosed by physical examination, magnetic resonance imaging (MRI), or arthroscopy. Since 2000 there has been a dramatic increase in MRI accessibility and in 2009, around, 50,000 MRI examinations of the knee were undertaken in Sweden, giving an incidence of about 5,5 per 1000 inhabitants [30]. OA, on the other hand, has traditionally been diagnosed mainly by clinical examination and X-ray. In this study knee OA was classified as patients being diagnosed with OA in specialized healthcare. This population is most likely patients with more severe OA then patients being diagnosed with OA in primary healthcare and not included in the study. Detection bias could be a limitation if reconstructed patients have a greater propensity to contact or be referred to specialist healthcare. However this is unlikely since patients treated non-surgically have the same risk to be diagnosed with OA in specialized during the first years of follow-up. Based on the register data, it is not possible to define if the knee diagnosed with OA is the same knee that had previous CL injury, although it has been shown that this is usually the case. For example van der Hart et al. showed that there is an almost 50% higher prevalence of OA in the injured knee [31]. Another limitation is that patients diagnosed or treated in outpatient setting before 2001 are not included in the study. This explains why the descriptive results presented in this study are not coherent with the results presented earlier [3]. This could potentially cause some bias as the inpatient cases might be severe cases. However, restricting the results to those diagnosed after 2001 did not change the results notably. Another limitation is that patients with CL injuries, who never seek medical care for their injury, are not included in this study. However since CL injuries lead to a rapid hemarthrosis of the knee precluding continuation of activity [32], most patients are likely to visit a health-care provider where a correct diagnosis can be established. It is a limitation that bilateral injuries and the type of CL injury cannot be identified, making it impossible to differ between ACL injury and PCL injury. Data from the Swedish Cruciate Ligament Register show that 2% of the patients underwent bilateral reconstruction [33]. Isolated posterior cruciate ligament injuries are uncommon and account for an estimated 3% of all acute knee injuries [34]. Thus, the vast majority of our patients represent patients with ACL injuries and the results can be extrapolated to all ACL injuries. Like most chronic diseases, OA is complex and multifactorial. It cannot be excluded that there might be differences between the patients treated with CL-R and those treated non-operatively that we did not have information on, e.g. level of physical activity.

The increasing number of older people and the changes in lifestyle throughout the world mean that the burden of musculoskeletal injuries and diseases will increase dramatically [1]. Our study reports a prevalence of OA of 10% in patients between 15–60 years 10 years after they were diagnosed with a CL injury. This is less than reported in earlier studies which is most likely explained by the fact that the definition of OA in this study was based on hospital data and concomitant registered diagnosis of OA in specialist care. Patients only diagnosed with OA in primary were not identified in this study.

In this study 41% of the patients had a diagnosed meniscal injury which is to be compared with 25–65% described in earlier studies [17]. Meniscal injury cannot however be assessed as a dichotomy, it is a continuous variable ranging from traumatic lesions to degenerative injuries. Further, meniscal injuries are not always symptomatic [35]. Since we were interested in the acute traumatic injuries, we attempted to exclude degenerative meniscal injuries as well as meniscal injuries in patients older than 35 years. Our results showed that concomitant meniscal injury was the strongest risk factor to develop OA which is coherent with earlier results [14]. Results from cohort studies have shown an association been early CL reconstruction and fewer meniscal surgeries. Reducing the risk of meniscus tear would mean that CL-R might protect a CL injured knee from post-traumatic OA. Although some cohort studies support this hypothesis there are conflicting results reporting no difference or even an increased risk for OA [14], [25], [36]. A randomized clinical trial by Frobell et al did not show a difference in risk for radiographic OA up to 5 years in patients treated surgically compared to those treated non-surgically [27], [37]. Our results suggest that there is an increased long term risk for OA after CL-R. Taking into account the more recent published results together with ours we conclude that decreasing the long-term risk for post-traumatic OA after CL injury is not an argument for CL-reconstruction.

## Conclusions

CL reconstructive surgery did not show a protective effect on knee OA in the long term.
